# Clinical feasibility of atlas‐based auto‐segmentation for organ‐at‐risk contouring in head‐and‐neck radiotherapy

**DOI:** 10.1002/acm2.70150

**Published:** 2025-07-13

**Authors:** Han Liu, Benjamin Sintay, David Wiant

**Affiliations:** ^1^ Department of Radiation Oncology Cone Health Cancer Center Greensboro North Carolina USA

**Keywords:** auto‐segmentation, knowledge‐based planning

## Abstract

**Purpose:**

Head‐and‐neck (HN) contouring presents significant challenges due to the complex anatomy of the region and the proximity of organs‐at‐risk (OARs) to the target. Manual contouring is time‐consuming, labor‐intensive, and prone to inter‐ and intra‐observer variability. Additionally, contours delineated prior to treatment may not accurately reflect the patient's anatomy over the extended treatment course. This study explores the feasibility of using auto‐generated OAR contours in HN planning.

**Methods:**

A retrospective study was conducted on 20 patients, each with a planning CT and 35 CBCT images. OARs were manually delineated and automatically generated using atlas‐based segmentation algorithms on planning CTs. Treatment plans were created via a novel two‐step optimization process, incorporating a knowledge‐based planning solution for both auto‐generated (aOAR‐plan) and manual OARs (mOAR‐plan). The accuracy of auto‐generated contours was quantified using the overlap index (*OI*) and dose similarity coefficient (*DSC*). Planning dose comparisons were performed between aOAR‐ and mOAR‐plans. Additionally, planning doses were transferred from CT to CBCTs based on clinical shifts, and contour‐based deformable registration was employed to calculate cumulative doses. Cumulative dose evaluations were performed for serial organs and parallel organs that can be fully imaged within the CBCT field.

**Results:**

For OARs located farther from the target, even though atlas‐based segmentation could not accurately reproduce patient anatomy, excellent agreement in planning doses was observed between the aOAR‐ and mOAR‐plans. The average *OI*/*DSC* between manual and auto‐generated contours were 85.0% ± 5.4%/87.4% ± 2.6% for the larynx, 76.0% ± 9.3%/77.0% ± 5.8% for the pharynx, 89.9% ± 4.0%/87.8% ± 2.5% for the oral cavity, 81.5% ± 10.5%/78.2% ± 5.9% and 83.2% ± 10.6%/77.8% ± 7.5% for the left and right parotid, respectively. The cumulative dose differences for OARs between aOAR‐ and mOAR‐plans were within 2 Gy for 90% of patients studied.

**Conclusion:**

Automated‐contouring tools offer improvement in contour consistency, provide acceptable doses compared with manually drawn contours in HN radiotherapy.

## 1 INTRODUCTION

Head‐and‐Neck (HN) cancer ranks as one of the most common cancers globally, with approximately 1.1 million new cases diagnosed annually.^1^ Radiotherapy has long been a cornerstone of treatment, either as a primary approach or as an adjuvant to surgical resection, to treat HN cancer patients over the past few decades.^2^, ^3^ The complexity of maintaining quality‐of‐life post‐treatment is influenced by factors such as the location of the tumor, disease stage, and extent of the disease. Studies indicate that over 90% of HN patients undergoing radiotherapy suffer some extent of side effects, such as xerostomia, oral discomfort, and so forth. ^4^, ^5^, ^6^ Despite recent advancements in delivery technologies, including intensity modulated radiotherapy (IMRT)^7^ and volumetric modulated arc therapy (VMAT),^8^ which offer highly conformal dose and very sharp dose fall‐off around the target, treatment planning for HN cancer remains extremely challenging. This difficulty arises not only from the location and size of the tumor, but also from the proximity of over 30 critical structures near to target area. The primary objective in treatment planning is to ensure adequate target coverage while minimizing radiation exposure to surrounding organs‐at‐risk (OAR) and normal tissues.

Target and OARs contouring has consistently been identified as one of the highest‐risk step in the treatment planning process, with accurate delineation has been considered essential for successful outcome.^9^ However, there are significant challenges with manual contouring in clinical practice: personnel costs account for up to 30% of the total radiotherapy treatment cost per patient; contouring errors pose substantial risks; and OAR delineation remains a major source of variability, and so forth.^10^, ^11^ Study of radiotherapy timelines have showed that the time required for contouring is comparable to that needed for creating treatment plans for HN cancer patients,^12^ which highlights the time‐consuming and labor‐intensive nature of the process. Additionally, inter‐, and intra‐observer variabilities further complicate the consistency and accuracy of manual contouring process. A failure mode and effects analysis (FMEA) conducted by AAPM task group #100 identified that four of the top ten most hazardous steps in radiotherapy, as ranked by average risk priority numbers, stem from errors in target and critical structure delineation.^9^

Semi‐automatic and fully automated contouring software are increasingly gaining traction in routine clinical practice. Compared to manual delineation, automated methods improve consistency, save time, and greatly reduce the chances of gross errors. However, practices have been slow to adopt automated techniques, often citing contour accuracy concerns as a limiting factor. In HN planning, acceptance of these tools has been particularly slow, primarily due to discrepancies between “expert” manual and automated contours. Much effort has been made to assess the impact of contour uncertainties, including inter‐ and intra‐observer uncertainties and automatic segmentation, on radiation treatment planning for HN patients.^13^, ^14^, ^15^ However, most attempts were limited to the planning phase alone. Given that the HN treatment course often spans several weeks and include multiple fractions, concerns arise that manual, human‐derived “expert” contours drawn on the planning CT prior to radiation treatment may not accurately represent the actual patient evolving anatomy over an extended course of treatment due to factors such as weight loss and daily anatomical changes, such that doses evaluated based on pre‐treatment contours may not represent the actual delivered doses. If not properly considered, these uncertainties could lead to target underdosing and/or excessive dosing to the surrounding normal tissues and critical organs, compromising treatment efficacy and patient safety.

Auto‐segmentation opens the door for a new era in treatment planning. This study assessed the clinical acceptability of delivered doses–defined as cumulative dose differences within 2 Gy for the maximum dose to serial organs and the mean dose to parallel organs–by comparing treatment plans generated from automatically segmented OAR contours against those created using manual delineations. If this is true, it may be feasible to use auto‐generated OAR contours for HN treatment planning.

## 2 METHODS AND MATERIALS

This study retrospectively reviewed data from 20 HN cancer patients treated between 2019 and 2021. All patient information was sourced from an institutional review board‐approved registry. Table 1 summarizes the demographics and clinical characteristics of the patient cohort. The clinical treatment involved three prescribed dose levels: 70 Gy delivered in 35 fractions to the primary tumor, 56 Gy to the low‐risk nodal region, and a simultaneous integrated boost (SIB) of 63 Gy to areas with macroscopic disease. The primary treatment planning objective was to ensure that at least 95% of each planning target volume (PTV) received the prescribed dose.

**TABLE 1:** Patient demographics and clinical characteristics.

Patient #	Tumor site	Stage	*V*(PTV_High_) (cc)	*V*(PTV_Med_) (cc)	*V*(PTV_Low_) (cc)
1	Tongue	IVA (cT1, cN2b, cM0)	24.6	199.3	492.8
2	Oropharynx	IVA (cT2, cN2c, cM0)	177.3	407.3	146.3
3	Oropharynx	III (cT4, cN1, cM0)	243.9	243.7	364.4
4	Oropharynx	I (cT2, cN1, cM0)	113.8	151	374.7
5	Oropharynx	I (cT2, cN1, cM0)	225.2	269.9	495
6	Laryngopharynx	IVA cT1 cN2b cM0	275.8	499.7	207.6
7	Oropharynx	I (cT2, cN1, cM0)	233.8	333.2	583.7
8	Laryngopharynx	IVB (cT2, cN3b, cM0)	318.6	475.7	220.8
9	Nasopharynx	III (cT1, cN2, cM0)	30.9	189.7	334.7
10	Laryngopharynx	IVA (cT3, cN2c, cM0)	243	539.7	317.1
11	Larynx	III (cT3, cN0, cM0)	106	178.4	432
12	Tonsil	II (cT3, cN2, cM0)	256.4	532.3	380.9
13	Tonsil	II (cT2, cN2, cM0)	217.6	668.9	258
14	Tonsil	II (cT3, cN2, cM0)	237.6	406.1	313.9
15	Larynx	IVA (cT4a, cN1, cM0)	137.3	326.1	373.6
16	Larynx	III (cT1, cN1, cM0)	56.9	111.4	301.4
17	Larynx	II (cT2, cN0, cM0)	80.8	165.3	388.6
18	Larynx	II (cT2, cN0, cM0)	108.1	129.5	302.3
19	Tonsil	I (cT2, cN1, cM0)	162.1	176.8	438.7
20	Larynx	III (cT3, cN0, cM0)	38.3	82.9	319

All patients included in this study underwent a planning computed tomography (CT) scan and at least 35 cone‐beam CT (CBCT) scans for daily setup verification. The reference planning CTs were obtained with a field of view large enough to encompass the head and neck region. The imaging parameters included 120 kVp and 195 mAs, with a voxel size of 1.17 × 1.17 × 2 mm. The daily CBCT protocol utilized full‐fan and half‐trajectory modes, with imaging parameters of 100 kVp and 150 mAs, and a voxel size of 0.51 × 0.51 × 1.99 mm. All OARs were manually delineated and automatically generated using MIM (MIM Vista, Cleveland, OH) atlas‐based segmentation algorithms on the planning CT image. The contour atlas was developed using data from 80 previously treated HN cancer patients that were representative of the diagnosis of our patient population, specifically patients diagnosed with cancers involving the larynx, tonsil, laryngopharynx, nasopharynx, oropharynx, or tongue in our clinic. A customized MIM workflow was developed to clean up all the contours and create planning PTVs, uninvolved OAR structures, and auxiliary structures such as ring and post‐avoidance regions. Treatment plans were developed using a novel semi‐automated two‐step optimization approach leveraging knowledge‐based planning techniques for both auto‐generated OARs (aOAR‐plan) and manually delineated OARs (mOAR‐plan) structure sets.^16^ To isolate the impact of auto‐generated OARs on the radiation treatment process, identical manually defined target volumes (all three PTVs) were used for both aOAR‐ and mOAR‐plans. Geometric and dosimetric evaluations were conducted, with geometric accuracy assessed using the overlap index (*OI*) and dose similarity coefficient (*DSC*). The *OI* measures the similarity between the mOAR and aOAR contours by calculating the ratio of the volume of their intersection to the volume of the mOAR contour, and is defined as:

$$OI=\frac{Volume\left(mOAR\right)\;\cap \;Volume\left(aOAR\right)}{Volume\left(mOAR\right)}\;.$$

The dice similarity coefficient is calculated as:

$$DSC=\frac{2*Volume\left(mOAR\right)\;\cap \;Volume\left(aOAR\right)}{Volume\left(mOAR\right)\cup \;Volume\left(aOAR\right)}\;,$$

where ∩ and ∪ are the intersection and union of aOAR and mOAR contours, respectively. The *OI* and Dice similarity coefficient range from 0 to 1, where 1 indicates a perfect match and 0 signifies no overlap. Dosimetric comparisons evaluated differences between the doses generated for aOAR‐ and mOAR‐plans. The dosimetric indices used for plan comparisons include the maximum dose for the mandible, brainstem, and spinal cord, and the mean dose for the larynx, pharynx, oral cavity, and left and right parotid glands.

**FIGURE 1:**
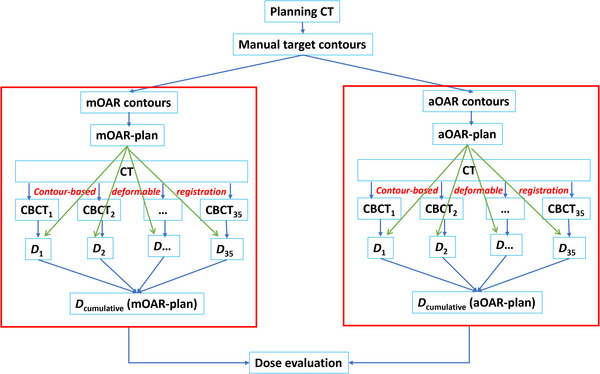
Flow diagram of the overall workflow.

Due to the limited field‐of‐view in daily CBCT images, only fully visible OARs were manually delineated on each daily CBCT image set for cumulative dose analysis. The list of structures were serial organs: mandible, brainstem, and cord; and parallel organs: parotid glands, oral cavity, larynx, and pharynx. Planning doses from both the aOAR‐ and mOAR‐plans were transferred from the planning CT to the daily CBCTs based on recorded clinical shifts. Contour‐based deformable registrations were applied to calculate the cumulative doses for all OARs. The differences in cumulative dose for serial and parallel organs served as indicators of agreement between the aOAR‐ and mOAR‐plans. Figure 1 provides a flow diagram outlining the overall workflow of the study.

## 3 RESULTS

**FIGURE 2:**
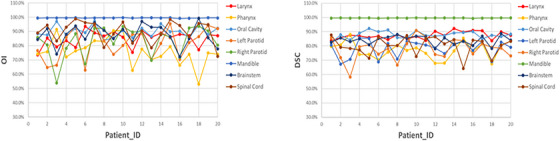
*OI* and Dice Similarity Coefficient (*DSC*) comparing manual and automatically generated OAR contours. OAR, organs‐at‐risk; *OI*, Overlap Index.

Geometric evaluations using the *OI* and *DSC* were conducted for OARs, comparing auto‐generated and manually drawn contours, as shown in Table 2 and Figure 2. Satisfactory geometric agreement was achieved for all key OARs, including the mandible, brainstem, spinal cord, larynx, pharynx, oral cavity, and left and right parotid glands, forming the foundation for this study.

**TABLE 2:** *OI* and dose similarity coefficient (*DSC*) between the manual and auto‐generated OAR contours.

	*OI*	*DSC*
**Mandible**	99.4 ± 0.1%	99.6 ± 0.1%
**Brainstem**	87.6 ± 7.3%	84.1 ± 3.1%
**Spinal Cord**	87.7 ± 7.3%	79.2 ± 8.0%
**Larynx**	85.0 ± 5.4%	87.4 ± 2.6%
**Pharynx**	76.0 ± 9.3%	77.0 ± 5.8%
**Oral Cavity**	89.9 ± 4.0%	87.8 ± 2.5%
**Lt Parotid**	81.5 ± 10.5%	78.2 ± 5.9%
**Rt Parotid**	83.2 ± 10.6%	77.8 ± 7.5%

Abbreviations: OAR, organs‐at‐risk; *OI*, Overlap index.

**FIGURE 3:**
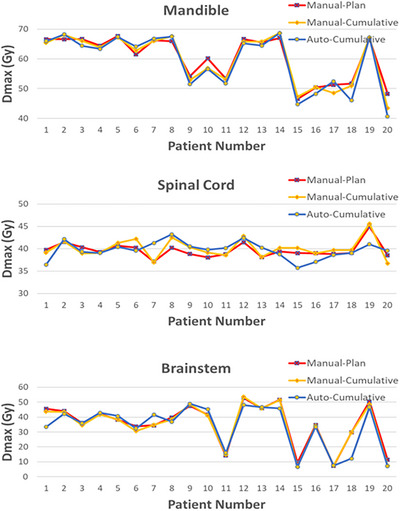
Dose comparisons for planning doses of mOAR‐plans, and cumulative doses of aOAR‐ and mOAR‐plans for serial OARs. aOAR‐plans, auto‐generated OARs; mOAR‐plans, manually delineated OARs; OAR, organs‐at‐risk.

**FIGURE 4:**
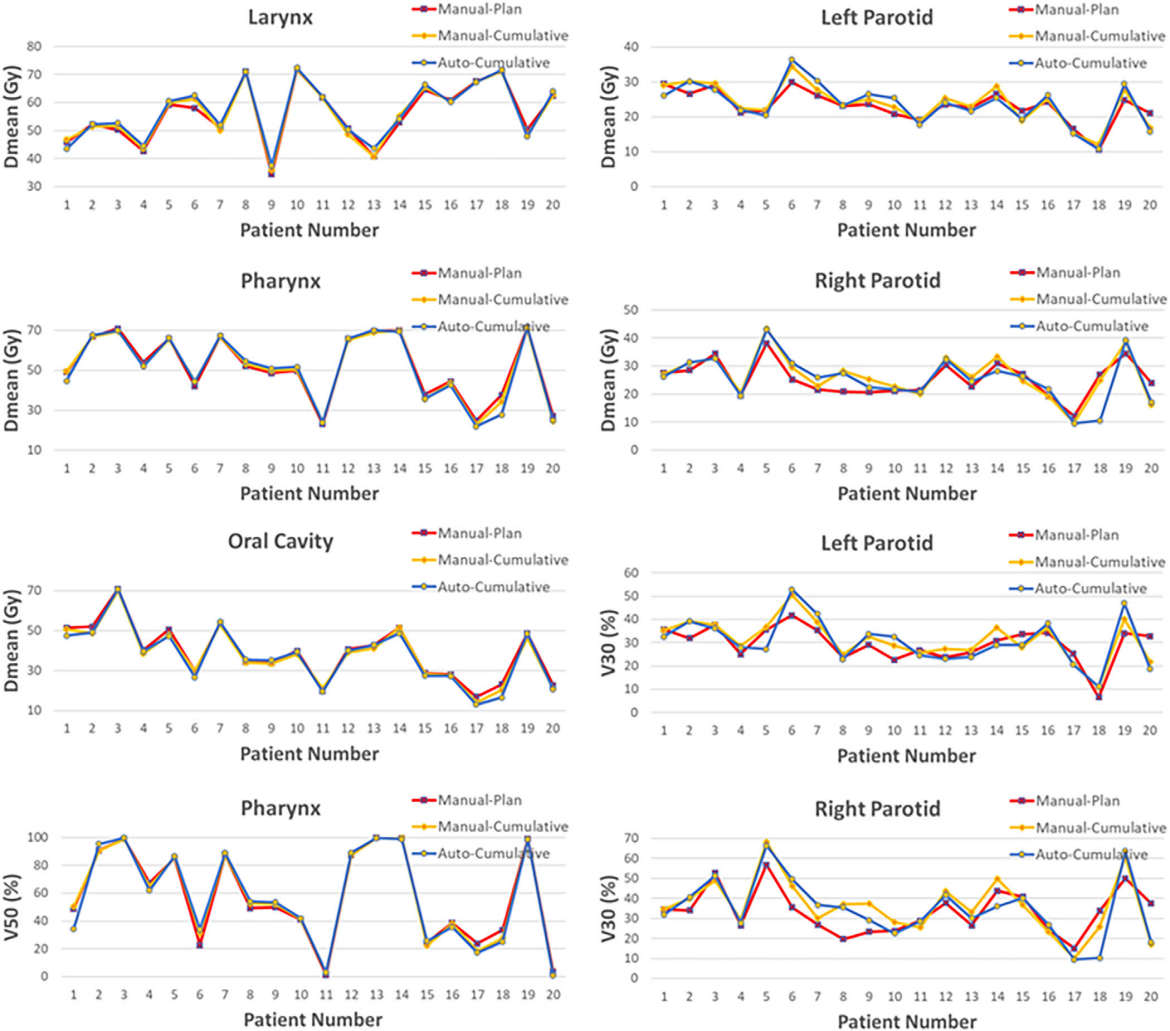
Dose comparisons for planning dose of mOAR‐plans, and cumulative doses of aOAR‐ and mOAR‐plans for parallel OARs. aOAR‐plans, auto‐generated OARs; mOAR‐plans, manually delineated OARs; OAR, organs‐at‐risk.

Figures 3 and 4 present mOAR‐plans doses, as well as the cumulative doses for both aOAR‐ and mOAR‐plans for serial and parallel organs, respectively. No marked cumulative dose differences for OARs were observed between the aOAR‐ and mOAR‐plans.

**FIGURE 5:**
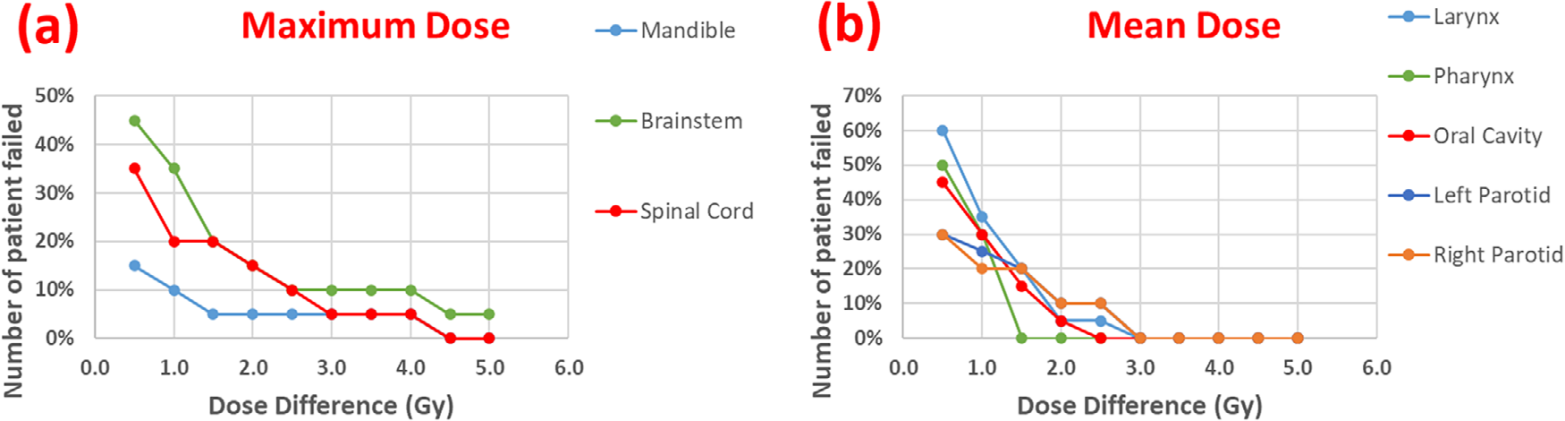
Percentage of patients with cumulative dose differences exceeding a set criterion between mOAR‐ and aOAR‐plans. (a) Maximum dose differences for serial organs; (b) Mean dose differences for parallel organs. aOAR‐plans, auto‐generated OARs; mOAR‐plans, manually delineated OARs.

In this study, cumulative dose differences were used as an indicator of the agreement between plans generated from automated and manual contours. Figure 5 illustrates the percentage of patients with cumulative dose differences greater than a predefined threshold between the mOAR‐ and aOAR‐plans for both serial and parallel organs. The results show good agreement between the two plans for the majority of patients studied. For 90% of the patients, the cumulative dose differences—measured as the maximum dose for mandible, brainstem, and spinal cord, and the mean dose for the larynx, pharynx, oral cavity, and left and right parotid glands—were within 2 Gy.

## 4 DISCUSSION

Contouring accuracy is a critical component of HN radiotherapy treatment planning. Accurate delineation of targets and OAR is essential to ensure that the prescribed radiation is precisely delivered to the target area while sparing the surrounding healthy tissues and critical structures, ultimately influencing both patient survival and quality of life. Over the past two decades, significant research has focused on improving the accuracy of contouring, including advancements in imaging technologies for enhanced image resolution and contrast, the development of consensus contouring guidelines, and the integration of artificial intelligence and machine learning algorithms to automate contouring to reduce the inter‐ and intra‐observer variability, and so forth. Various geometric metrics, such as *OI*, *DCS*, and centroid distance, have been proposed to assess contouring accuracy. However, those metrics fall short in clinical relevance because they do not incorporate dosimetric information and fail to account for the relative relationship between the target and OARs. Recent efforts have also explored whether accurately contouring salivary and swallowing structures is essential for sparing them in HN VMAT plans.^15^ A limitation of such study is that it only focuses on the treatment planning phase, without considering anatomical changes that may occur throughout the course of treatment. Given that atypical HN radiotherapy course consists of 35 fractions, contours drawn on a planning CT image may be less robust than anticipated since they do not account for anatomical changes over time, leading to potential discrepancies between the planned and actual delivered doses.

Uncertainty in the radiation dose delivered to tumors during EBRT arises from various factors. The International Commission on Radiation Units and Measurement (ICRU) recommends that the dose be delivered to within ± 5% of the prescribed dose. This brings up the question of how precise the contours must be in the radiotherapy treatment planning process to achieve this level of accuracy.

In this study, we aimed to evaluate the feasibility of using automated OAR contours for HN treatment planning and assess their impact on dosimetry by comparing the aOAR‐plan with the mOAR‐plan. Reasonable agreement was observed between the manual and auto‐generated contours for most OAR structures. It is important to note that atlas‐based segmentation was employed to obtain the auto‐generated contours in this study. With the rapid development of artificial intelligence/machine learning‐based algorithms, we anticipate that auto‐generated contours will increasingly align with manually drawn contours while providing enhanced consistency. Therefore, over time, the results from this study could improve as these technologies evolve. Additionally, the newly developed two‐step optimization method played a crucial role in this evaluation, as it enables the generation of unbiased high‐quality plans while reducing inter‐planner variability based on a set of given structure sets.^16^

We acknowledge that clinical alignment between daily CBCT and planning CT involves both translational and rotational components. Translating the dose volume from the planning CT to the CBCT does not alter the dose values; however, rotating the volume can introduce interpolation errors that may affect dose accuracy. In our clinical practice, a maximum rotational adjustment of 2 degrees is permitted during the daily CBCT. Even within this limit, the rotation could potentially smooth out dose hot spots if the rotation is significant enough. It is important to note that the transferred dose serves only as an estimate of the delivered dose during daily treatment.

In this study, differences in cumulative doses for the OARs were used as an indicator of agreement between the aOAR‐ and mOAR‐plans, with contour‐based deformable registration employed to obtain the cumulative dose. The following contours were delineated on each CBCT images to perform contour‐based deformable registration: mandible, brainstem, cord, parotid glands, oral cavity, larynx, and pharynx. Good dosimetric agreement between the aOAR‐ and mOAR‐plans were observed for both serial (Figure 3) and parallel organs (Figure 4). For 90% of patients, the cumulative dose differences between the aOAR‐ and mOAR‐plans were within 2Gy, indicating that automated contours to effectively replace manual contours without significantly compromising plan quality.

Caution must be exercised when using auto‐generated contours for serial organs that are near the PTV, especially in the high‐risk PTV regions, as the maximum dose to these organs is a critical factor in the planning process. Some level of manual editing of automated serial organ contours may be required on a case‐by‐case basis, particularly for CT slices close to the high‐risk PTV volume. Due to the large variation of brachial plexus anatomical position in the atlas training set and noticeable inter‐observer contour uncertainty, the atlas‐based segmentation failed to generate the brachial plexus satisfactorily. Due to the significant impact of inaccurate delineation of the brachial plexus on plan quality, manual contour was used for the brachial plexus in both the aOAR‐ and mOAR‐plans.

Our ongoing efforts involve evaluating AI‐based contouring tools available on the market and integrating automation into the clinical treatment planning process for the HN treatments, including auto contour generation for all OARs and helper structures. Additionally, we are continuing to develop Eclipse scripts that automate the use of RapidPlan to adjust the dose objective in the second step of the optimization process.

We also want to point out that this is a retrospective study evaluating whether auto‐generated OAR contours are sufficiently accurate for HN treatment planning. The impact on clinical outcome, which is of greater clinical relevance, falls outside the scope of this study and could be addressed in future clinical trials.

## 5 CONCLUSIONS

Manually delineated OAR contours are used to represent patient anatomy throughout long courses of radiation treatment. However, these contours often fluctuate by several millimeters on a daily basis for HN patients, which raises questions about the time and effort required for precision delineation. Automated tools can generate contours more quickly, offer improvement in contour consistency, and provide clinical acceptable delivered doses when compared to manual contours for HN patients.

## AUTHOR CONTRIBUTION

**Han Liu**: designed the analysis, collected the data, performed the analysis, and drafted the manuscript. **Benjamin Sintay**: assisted with the study design and drafted the manuscript. **David Wiant**: assisted with the study design and drafted the manuscript.

## CONFLICT OF INTEREST STATEMENT

B. Sintay and D. Wiant are co‐founders of Fuse Oncology.

## Data Availability

Authors are not able to share data at this time.
